# Exercise interventions for pulmonary function impairment: rehabilitation strategies from the acute phase to long-term sequelae

**DOI:** 10.3389/fphys.2025.1650078

**Published:** 2025-09-24

**Authors:** Yunpeng Guo, Lei Huo, Zhanyi Zhang

**Affiliations:** ^1^ School of Physical Education, Henan Normal University, Xinxiang, China; ^2^ Zhengzhou Tourism College, Zhengzhou, China

**Keywords:** pulmonary function impairment, exercise intervention, respiratory rehabilitation, cardiopulmonary adaptation, personalized exercise prescription

## Abstract

Pulmonary function impairment, as a major global public health concern, significantly affects respiratory function, quality of life and mortality. Its pathological features include impaired gas exchange, reduced exercise tolerance, and chronic inflammatory responses. Conventional non-pharmacological interventions, such as protective mechanical ventilation, prone positioning, and extracorporeal membrane oxygenation (ECMO), can improve oxygenation but are limited by risks of complications, poor patient tolerance, and high dependence on equipment. Pharmacological therapies may regulate inflammatory responses; however, their long-term application is constrained by safety concerns and interindividual variability in efficacy. The discussion of the benefits and mechanisms of exercise interventions includes an explanation of their applicability to patients across various types and stages of pulmonary function impairment. In addition, this study systematically elucidates the mechanisms and implementation strategies of exercise interventions across different stages of pulmonary impairment, including the acute phase, recovery phase, and long-term management phase. It further proposes a personalized exercise prescription framework based on biomarkers. The study emphasizes the development of dynamic adjustment systems and stratified management strategies tailored for special populations such as the elderly and children, thereby providing a theoretical foundation for optimizing pulmonary rehabilitation outcomes.

## 1 Introduction

Impairment of lung function has emerged as a significant focus in clinical research. It can result in acute lung injury and potentially long-term functional impairment through various pathological mechanisms (Cheon et al., 2021; Cha et al., 2024; Ivanova et al., 2023), including direct tissue invasion (Shetty and Idell, 2023; Narasaraju et al., 2024), excessive inflammatory responses (Montazersaheb et al., 2022), hypoxic injury (Swenson and Hardin, 2023), and airway dysfunction (Gai et al., 2023). Current clinical interventions, including pharmacotherapy, oxygen therapy, and mechanical ventilation, present several limitations: pharmacological treatments often entail dose-dependent side effects (Schols et al., 2001); oxygen therapy may restrict patients' mobility (Cani et al., 2019); and mechanical ventilation carries the risk of iatrogenic complications such as barotrauma (Gammon et al., 1992). In contrast, exercise intervention has demonstrated unique advantages. Its safety, cost-effectiveness, and non-invasive nature offer a promising alternative for pulmonary function rehabilitation (Garvey et al., 2016).

Pulmonary function impairment may evolve from an acute phase, characterized by severe inflammation and respiratory distress (Bos and Ware, 2022), to chronic or long-term sequelae involving persistent ventilatory limitations (Herridge et al., 2011), reduced exercise capacity, and impaired quality of life. Therefore, developing intervention strategies that address the entire continuum from the acute phase to long-term sequelae is essential for comprehensive pulmonary rehabilitation. The clinical efficacy of exercise intervention in pulmonary function rehabilitation has been supported by evidence-based studies (Fiorentino et al., 2020). Through multiple physiological mechanisms—including pulmonary function optimization, inflammation suppression, regulation of oxidative stress, and enhancement of exercise tolerance—exercise intervention significantly improves both the physical and psychological states of patients (Pleguezuelos et al., 2023; Xiong et al., 2023; Liu et al., 2023; Jiang et al., 2023).

Compared to traditional pharmacological and passive treatments, this proactive rehabilitation approach offers distinct non-pharmacological, cost-effective advantages with improved functional outcomes (Incorvaia et al., 2019). Despite advances in conventional pulmonary therapies, several challenges remain in exercise intervention research: (1) incomplete understanding of molecular mechanisms driving lung tissue repair (Chen Y. et al., 2024); (2) fundamental differences between acute lung injury and chronic impairment requiring stratified treatment strategies (Xiong et al., 2023); (3) insufficient systematic evidence on optimal timing, dose-response, and individual variability of exercise interventions (van der Schoot et al., 2022). The lack of stage-specific exercise protocols often leads to suboptimal recovery in acute injury and may exacerbate long-term disability (Watz et al., 2014). Therefore, optimizing stage-specific and personalized exercise interventions across the acute to long-term sequelae phases is essential for advancing pulmonary rehabilitation.

This review integrates multidimensional research evidence to elucidate the scientific paradigm of exercise therapy in the full-spectrum management of pulmonary function impairment from a pathophysiological perspective The focus of this study includes: 1) Establishing differentiated intervention pathways for acute lung injury and chronic functional impairment stages based on disease staging characteristics; 2) Systematically clarifying the exercise dose-response of exercise intervention at various stages of the disease, with particular emphasis on the dynamic regulatory mechanisms during the acute stage of mild damage and the long-term sequelae phase; 3) Constructing a risk–benefit assessment system through evidence-based medicine, with a focus on analyzing the safety thresholds and risk warning indicators of intervention protocols. This summarizes available evidence and proposes potential considerations for exercise prescriptions that may inform clinical practice in precision pulmonary rehabilitation.

The innovation of this research lies in: Unlike existing reviews, which often focus on either acute or chronic stages in isolation, this review adopts a full-cycle perspective—spanning acute lung injury to long-term sequelae—and incorporates biomarker-informed stratification to tailor exercise interventions. In addition, it addresses the unique considerations for exercise interventions in special populations, a dimension often overlooked in previous literature. This integrative approach not only synthesizes current evidence but also proposes a practical model for precision rehabilitation.

## 2 Methods

This narrative review synthesizes evidence on exercise interventions for pulmonary function impairment across the continuum from acute to chronic stages. A comprehensive PubMed search was conducted from database inception to 1 May 2025, using both Medical Subject Headings (MeSH; e.g., “Pulmonary Disease, Chronic Obstructive”) and free-text terms (e.g., “pulmonary rehabilitation,” “exercise intervention”), combined with Boolean operators, without language restrictions. The search strategy was supplemented by manual screening of reference lists to identify additional relevant studies. Eligible conditions included pulmonary infections—typically defined as pneumonia caused by bacterial, viral, or other pathogenic agents and often associated with acute pulmonary function impairment—chronic obstructive pulmonary disease (COPD), characterized by progressive airflow limitation and chronic airway inflammation, and interstitial lung diseases (ILDs), which involve diffuse inflammation and fibrosis of the lung parenchyma, leading to restrictive ventilatory defects. Asthma and cystic fibrosis were excluded owing to their distinct pathophysiological mechanisms. Peer-reviewed randomized controlled trials, cohort studies, and systematic or narrative reviews were prioritized. Evidence was narratively synthesized according to disease stage (acute, transitional, recovery, and long-term phases) and type of exercise intervention.

## 3 Intervention for acute pulmonary function injury

### 3.1 Definition and clinical characteristics of the acute phase

The concept of acute lung injury (ALI) was first introduced by Ashbaugh and colleagues in 1967 (Huidobro et al., 2021; Ashbaugh et al., 1967). The 2012 Berlin Definition redefined acute respiratory distress syndrome (ARDS) into mild (PaO2/FiO2 201–300 mmHg), moderate (101–200 mmHg), and severe (≤100 mmHg) categories, eliminating the term ALI to standardize diagnosis (Force et al., 2012), and it is now one of the primary diseases in intensive care units (ICU) (Le et al., 2020). Current diagnostic standards require a comprehensive evaluation of four key factors: the onset window (≤7 days), characteristic hypoxemia, bilateral pulmonary infiltrates on imaging, and the exclusion of cardiogenic pulmonary edema (Bernard et al., 1994). It is noteworthy that the pathophysiological mechanisms of ARDS involve multiple influencing factors, including etiological heterogeneity, clinical evaluation discrepancies, and the complexity of radiological interpretation (Sjoding et al., 2016), all of which interact to form a complex pathophysiological network (Confalonieri et al., 2017). Due to the heterogeneity of this condition, a specific biomarker system for effective risk stratification has yet to be established (Bos and Ware, 2022; Huffmyer and Groves, 2015).

### 3.2 Conventional intervention strategies

#### 3.2.1 Non-pharmacological intervention therapies

Non-pharmacological respiratory support therapies mainly include the following three strategies: 1) In protective ventilation strategies, airway pressure release ventilation (APRV) maintains alveolar recruitment and prevents alveolar collapse (Jain et al., 2016; Papazian et al., 2019). The technical advantage lies in its adaptive ventilation scheme based on dynamic monitoring of respiratory system compliance (CRS), which can significantly suppress the progression of ventilator-associated lung injury (Lutz et al., 2024; Rawal et al., 2018). 2) Prone positioning ventilation, as a standard therapy for ARDS, has been shown in clinical practice to improve oxygenation by optimizing the transpulmonary pressure gradient (Boesing et al., 2022; Papazian et al., 2022). The core mechanism involves positioning changes that promote recruitment of the dorsal lung regions while alleviating excessive inflation of the ventral alveoli (Liao et al., 2024). 3) Extracorporeal membrane oxygenation (ECMO), as a final respiratory support measure, replaces pulmonary gas exchange through an extracorporeal circulation device (Conrad et al., 2018; Peek et al., 2009). Its core physiological effects include increasing mixed venous oxygen content and reversing hypoxic pulmonary vasoconstriction, thus significantly reducing pulmonary arterial pressure and pulmonary vascular resistance (Ficial et al., 2021; Sud et al., 2024; Belletti et al., 2025; Balasubram et al., 2024; Tu et al., 2024). ECMO is primarily indicated for critically ill patients with refractory hypoxemia who fail to achieve adequate oxygenation despite optimal conventional mechanical ventilation strategies (Peek et al., 2009).

#### 3.2.2 Pharmacological intervention therapies

Common pharmacological interventions in clinical practice include the following: 1) Neuromuscular blocking agents (NMBA) exert their effects through selective neuromuscular junction blockade (O'Gara et al., 2015; Chuang et al., 2024), with the primary action of eliminating patient-ventilator asynchrony, thus significantly reducing the risk of ventilator-associated lung injury (VALI) (Torbic et al., 2021). 2) Corticosteroids primarily demonstrate immunomodulatory properties in the treatment of ARDS. Their main mechanism involves regulating the inflammatory response pathways, antagonizing the synthesis and release of pro-inflammatory mediators (Bernard et al., 1987). Several randomized controlled trials have confirmed that these drugs can shorten the duration of mechanical ventilation and improve oxygenation parameters (Lewis et al., 2019; Dequin et al., 2023; Villar et al., 2020; Meduri et al., 2016). 3) Inhaled nitric oxide (iNO), as a selective pulmonary vasodilator (Yu et al., 2019), improves oxygenation in ARDS treatment by optimizing the ventilation/perfusion ratio (Hartwig et al., 2023; Ozturk et al., 2016). Mechanistic studies have confirmed that its molecular action pathway involves the activation of soluble guanylate cyclase (sGC)-cyclic GMP (cGMP)-protein kinase G (PKG) signaling cascade, ultimately inducing pulmonary vascular smooth muscle relaxation (Albert et al., 2017; Dowell et al., 2017).

### 3.3 Feasibility of exercise intervention

#### 3.3.1 Objectives and benefits of exercise intervention

Exercise-mediated biochemical cascades regulate the levels of circulating inflammatory mediators and activate endogenous homeostasis maintenance mechanisms (de Barcellos et al., 2021). The mechanisms involve enhancing the activity of antioxidant enzymes such as superoxide dismutase (Nemmar et al., 2018), and achieving free radical clearance by regulating the balance of the oxidative/antioxidative system (Rajizadeh et al., 2023). Notably, a scientifically designed exercise program can yield multidimensional clinical benefits: it significantly improves respiratory muscle performance and optimizes the ventilation/perfusion ratio during the acute phase; enhances fibrinolytic system activity to reduce the risk of thrombosis; and improves microcirculatory perfusion, thereby effectively controlling the incidence of secondary complications (Rodriguez-Blanco et al., 2021; Espinoza-Bravo et al., 2023).

#### 3.3.2 Challenges of exercise intervention

The window period from transitioning from mechanical ventilation to spontaneous breathing is a critical stage for the gradual recovery of patients with acute lung injury. For patients who have been bedridden for extended periods and undergone mechanical ventilation, respiratory and skeletal muscle weakness may occur. During the transition from mechanical ventilation to spontaneous breathing, it is important to avoid a decline in blood oxygen levels and inadequate ventilation due to inappropriate exercise methods.

#### 3.3.3 Feasibility analysis of exercise intervention for acute lung injury

Exercise interventions provide comprehensive benefits in pulmonary rehabilitation, with mechanisms that encompass pulmonary tissue repair, respiratory muscle function reconstruction, and systemic inflammation regulation. In the acute phase of ARDS, passive mobilization or minimal bedside exercises are recommended under strict monitoring to prevent deconditioning, as active aerobic exercise may pose risks of desaturation (Amidei and Sole, 2013), structured aerobic training is generally not recommended during the acute phase. However, in clinically stable patients with adequate oxygenation (SpO_2_≥90% with support) and hemodynamic stability, very-low-intensity activities such as bedside cycle-ergometry at minimal resistance or short supervised walking with frequent rests may be cautiously trialed (Schweickert et al., 2009). Several issues must be carefully considered during implementation: the patient’s cardiopulmonary reserve function threshold (Neder, 2023), fluctuations in disease progression, and long-term adherence to the intervention. Therefore, it is recommended to use a comprehensive assessment system that integrates respiratory mechanics parameters (such as airway resistance and pulmonary compliance), oxygenation indices (PaO2/FiO2), and psychological status evaluation scales. This precision strategy can effectively balance the dynamic relationship between exercise stress and organ function reserve, minimizing the risk of secondary damage.

## 4 Intervention during the transition phase from acute phase to recovery phase

### 4.1 Markers for the transition from acute phase to recovery phase

The identification system for biomarkers of disease progression in lung injury requires integration of multidimensional evaluation indicators: 1) In monitoring inflammatory response thresholds, interleukin-6 (IL-6), a key pro-inflammatory cytokine (Montazersaheb et al., 2022; Butt et al., 2016; Xu et al., 2023), exhibits dynamic changes that reflect the waxing and waning of systemic inflammation. When serum IL-6 levels decline to approximately 24 pg/mL—a threshold reported in [specific condition, e.g., ARDS or COVID-19–related lung injury] — it may serve as a predictive marker for inflammation resolution in similar pathological contexts (Majidpoor and Mortezaee, 2022), although this threshold has not been validated for other etiologies and should be interpreted with caution. IL-6 induces the synthesis of C-reactive protein (CRP) via the JAK-STAT signaling pathway (Kocak Tuf et al., 2021), thereby functioning as a systemic biomarker for evaluating the resolution of pulmonary inflammation (Agassandian et al., 2014).2)According to the stage-transition model established on the Berlin Definition, a persistent PaO_2_/FiO_2_ ratio above 200 mmHg may indicates a transition from moderate/severe ARDS to the recovery phase (Fan et al., 2018; Camporota et al., 2022). However, this is an extrapolation beyond the original diagnostic criteria. 3)In imaging evaluation, high-resolution computed tomography (HRCT) quantitative analysis reveals that a reduction of more than 40% in ground-glass opacity volume (Solomon et al., 2021) is significantly associated with the restoration of alveolar epithelial integrity (Lee et al., 2014; Milos et al., 2021), though its generalizability to other lung injury etiologies requires further study. The dynamic monitoring of the aforementioned biomarkers may provide an objective and quantifiable basis for clinical staging transitions.

### 4.2 Principles of exercise intervention management during the transitional window period

The precise identification and intervention strategy construction for the Transitional Window Phase should be based on a multimodal biomarker monitoring system. This phase is defined as a critical period during which the patient transitions from the acute inflammatory storm phase to the homeostatic recovery phase, with its core characteristic being the marginal restoration of organ functional reserve capacity. Clinical practice recommends the use of a “stepwise progression of metabolic equivalents (METs)” model, employing cardiopulmonary exercise testing (CPET) to continuously monitor the dynamic changes in the anaerobic threshold (AT) and respiratory compensation point (RCP) (Force et al., 2007), thereby establishing a dose titration model based on physiological limit thresholds.

The implementation of specific strategies should adhere to the principles of pathological classification: 1) For patients with chronic obstructive pulmonary disease (COPD), low-intensity walking training is recommended, applying a weekly 10% incremental scheme in exercise duration. Simultaneous monitoring of SpO_2_ fluctuation amplitude (Δ ≤ 4%) and the gradient of transcutaneous carbon dioxide pressure (PtCO_2_) is advised. 2) During the endurance training phase of acute respiratory distress syndrome (ARDS) recovery, a progressive load adjustment method is recommended. Training intensity should be regulated based on the Borg Rating of Perceived Exertion scale (scores 4–6), utilizing a nonlinear incremental pattern with weekly increases of 5%–8% in load (O’Bryan et al., 2022). 3) In selected patient populations with adequate baseline functional capacity and clinical stability, high-intensity interval training (HIIT) protocols may be considered (Gibala et al., 2012; Robinson et al., 2017). Evidence suggests that, by enhancing mitochondrial biogenesis efficiency, appropriately tailored HIIT can achieve greater improvements in ventilatory capacity within the same training duration compared to continuous exercise (Wen et al., 2019). All protocols should incorporate real-time biofeedback mechanisms, with a particular focus on monitoring the pulmonary stress index and dynamic changes in transpulmonary pressure, to ensure precise alignment between exercise-induced stress and organ functional capacity.

## 5 Rehabilitation intervention strategies for pulmonary function impairment

### 5.1 Definition and goals of rehabilitation

The recovery phase of pulmonary function impairment refers to the physiological remodeling process characterized by tissue repair and functional reconstruction following the acute injury stage (Yang et al., 2024). This phase is biologically marked by the resolution of systemic inflammation, restoration of the alveolar epithelial barrier, and gradual recovery of pulmonary ventilation and diffusion capacity. The core objective of clinical rehabilitation during this stage is to prevent secondary organ dysfunction, focusing on two main aspects: 1) Inhibiting disuse atrophy of the respiratory musculature through neuromuscular activation training (Levine et al., 2008); 2) Establishing a stepwise cardiopulmonary reconditioning protocol to counteract circulatory deconditioning caused by prolonged bed rest.

Studies have shown that more than 2 weeks of bed rest can result in a 10%–15% loss in skeletal muscle mass per week and a 20%–30% reduction in circulatory volume over a similar period (Kortebein et al., 2008; Pavy-Le Traon et al., 2007). These pathological changes significantly impair the efficiency of ventilation–perfusion matching, leading to a vicious cycle of restrictive ventilatory dysfunction and impaired diffusion capacity.

### 5.2 Disease-specific interventions

Patients in the pulmonary function recovery phase commonly exhibit persistent fatigue syndrome and cognitive impairments (Klinkhammer et al., 2024; Ceban et al., 2022; Global Burden of Disease et al., 2022; Hu et al., 2024; Taquet et al., 2024). Therefore, the primary focus of intervention during this phase is to enhance patients' physical fitness through fatigue management and progressive exercise regimens.

#### 5.2.1 Exercise superimposition therapy combined with non-invasive ventilation (NIV)

In the recovery or stable phase of chronic obstructive pulmonary disease (COPD), the rationale for NIV use shifts from acute respiratory support to adjunctive facilitation of physical rehabilitation (Coleman et al., 2019; de Medeiros Nogueira et al., 2022). The advantages of NIV over invasive mechanical ventilation include a significant reduction in the rate of intubation and the incidence of ventilator-associated pneumonia (VAP) (Squadrone et al., 2004; Popat and Jones, 2016). By providing bilevel positive airway pressure (BiPAP), NIV effectively counteracts intrinsic PEEP, improves ventilation/perfusion (V/Q) matching, and thus alleviates respiratory muscle fatigue (Sehgal et al., 2019). NIV helps facilitate lung ventilation and relieves patients’ dyspnea. Exercise therapy supported by NIV can assist patients in improving their exercise endurance and cardiopulmonary function. However, the evidence base for such applications remains limited, and findings in the acute care setting (e.g., reduced intubation rates or ventilator-associated pneumonia (VAP) incidence) cannot be directly extrapolated to the recovery phase. Further high-quality trials are needed to determine patient selection criteria, optimal ventilation settings, and long-term benefits in such settings.

#### 5.2.2 Synergistic application of flexibility training and oxygen therapy

Patients in the pulmonary rehabilitation phase often face clinical challenges such as impaired lung parenchymal elasticity and persistent dyspnea (Rajan et al., 2023). The synergistic application of flexibility training and oxygen therapy can aid patients with pulmonary fibrosis in addressing lung stiffness during the recovery phase and improving overall lung function. Moderate flexibility exercises can enhance lung mobility, promote alveolar expansion, and increase chest wall elasticity. Short-term use of oxygen therapy can improve blood oxygen saturation and exercise capacity in patients with idiopathic pulmonary interstitial fibrosis, reduce fatigue, and most importantly, alleviate dyspnea symptoms (Lin et al., 2024; Suzuki et al., 2020; Yanagita et al., 2024; Pagliaro et al., 2024).

### 5.3 Exercise intervention program during the rehabilitation period

Exercise training is an indispensable component of pulmonary rehabilitation (PR) (Maltais et al., 2014). Reducing physical activity and exercise during the recovery phase of pulmonary function impairment not only negatively impacts body functions beyond the lungs, but it can also exacerbate the inflammatory cascade in the lungs (Garcia-Aymerich et al., 2007) and it is essential to tailor exercise interventions according to the patient’s clinical stage and disease severity. The development of exercise intervention protocols for the recovery phase of pulmonary function impairment is crucial, aiming to help patients restore lung function, improve exercise endurance, and enhance muscle strength through moderate exercise training.

#### 5.3.1 Aerobic training

The biological effects of low-to-moderate intensity aerobic training in pulmonary rehabilitation have been validated by numerous studies. These effects include improvements in functional independence and immune modulation. Research has confirmed that scientifically designed programs can significantly reduce serum levels of IgE and IgG (Mohamed and Alawna, 2021; Camargo Hizume-Kunzler et al., 2017).

Aerobic exercise modalities such as treadmill walking, overground walking, cycling ergometer training, and swimming have been extensively investigated in the context of pulmonary rehabilitation (Nolan et al., 2025; Liu et al., 2025). These interventions are typically prescribed at 40%–60% of peak oxygen uptake (VO_2_peak) or 50%–80% of peak work rate (WR peak), with the goal of improving cardiopulmonary fitness while minimizing undue ventilatory burden (Maltais et al., 2014; Spruit et al., 2013). Evidence demonstrates that treadmill and overground walking not only improve 6-min walking distance (6MWD) but also enhance oxygen utilization efficiency (Coppoolse et al., 1999), contributing to better functional capacity in daily activities. Stationary cycling or cycle ergometer training has been shown to specifically target peripheral muscle endurance and delay the onset of exertional dyspnea (Pitta et al., 2004). In addition, swimming and aquatic-based aerobic exercise offer the advantages of reduced joint loading and hydrostatic pressure, which facilitate breathing mechanics and may be especially beneficial for elderly or mobility-limited patients (Song and Oh, 2022; Waller et al., 2016). Collectively, these traditional aerobic approaches are supported by strong clinical evidence and form the cornerstone of pulmonary rehabilitation programs recommended by international guidelines.

Alongside traditional aerobic exercises such as brisk walking, treadmill training, stationary cycling, swimming, and low-impact dance, Tai Chi contributes to improved cardiopulmonary function (Guo et al., 2016) and immune regulation (Xiong et al., 2023) an integrated approach that combines Tai Chi with conventional aerobic modalities may not only optimize physiological outcomes but also enhance long-term adherence and overall therapeutic effectiveness.

#### 5.3.2 Resistance training

Resistance training has a significant beneficial effect on patients during the recovery phase of chronic obstructive pulmonary disease (COPD). Upper limb resistance training can effectively improve patients' arm motor function, activities of daily living, and occupational performance (Calik-Kutukcu et al., 2017). A systematic training program that enhances the strength of upper limb muscles such as the deltoid and biceps brachii can reduce respiratory rate and subjective fatigue during arm-supported exercises, while simultaneously promoting improvements in respiratory function. Lower limb resistance training, which strengthens the quadriceps and gluteus maximus, can significantly enhance respiratory compensation mechanisms, increase blood oxygen saturation, and optimize peripheral circulation, resulting in more efficient delivery of oxygen and nutrients. Ultimately, this improves pulmonary gas exchange efficiency (Topcuoglu et al., 2024).

#### 5.3.3 Expiratory muscle-specific training

Expiratory muscle-specific training aims to strengthen the expiratory muscles, thereby improving cough efficacy, airway clearance, and overall respiratory function (Templeman and Roberts, 2020; Chen X. et al., 2024). Patients perform repeated forceful expirations against additional resistance, which increases the workload of the expiratory muscles and enhances their strength and endurance.

#### 5.3.4 Personalized exercise prescription

In designing exercise prescriptions for patients with pulmonary function impairment, many studies advocate for individualized adjustments guided by the FITT principle (Frequency, Intensity, Time, and Type) to optimize both the safety and effectiveness of exercise interventions (Garvey et al., 2020; Torres et al., 2023). For instance, the American Thoracic Society/European Respiratory Society (ATS/ERS) pulmonary rehabilitation guidelines (2013) (Spruit et al., 2013) explicitly recommend tailoring exercise frequency, intensity, and duration based on disease severity, exercise tolerance, and individual clinical characteristics, with a preferential focus on appropriate exercise modalities such as aerobic endurance training, resistance training, and respiratory muscle training. These guidelines underscore that the precise and personalized application of the FITT framework can maximize enhancements in cardiopulmonary function, muscular capacity, and overall quality of life, while concurrently mitigating exercise-associated risks.

## 6 Rehabilitation management of long-term sequelae

### 6.1 Clinical diagnosis and treatment dilemmas of irreversible pulmonary tissue structural damage

#### 6.1.1 Pathological characteristics of pulmonary fibrosis and emphysema

Pulmonary fibrosis, a progressive fibrotic lung disease (Koudstaal et al., 2023), is caused by a multifactorial mechanism involving genetic factors (Wang and Young, 2022), autoimmune factors (Odell et al., 2022), occupational exposures (Perrot et al., 2023), toxins (Bradley et al., 2021), and chronic respiratory diseases (Koudstaal et al., 2023). Pathologically, it meets the diagnostic criteria for interstitial lung disease (Wijsenbeek et al., 2022). The process of pulmonary fibrosis leads to alveolar damage, characterized by excessive fibroblast proliferation and collagen deposition, resulting in lung tissue sclerosis (Hesse et al., 2022).Emphysema, as one of the main phenotypes of chronic obstructive pulmonary disease (COPD), involves the core pathological mechanism of abnormal distal airspace dilation in the terminal bronchioles, accompanied by alveolar wall destruction. This ultimately reduces the elasticity of the distal airways, leading to hyperinflation and impaired pulmonary gas exchange (Martin et al., 2013).

#### 6.1.2 Systemic complications of pulmonary function impairment

##### 6.1.2.1 Skeletal muscle atrophy and metabolic dysfunction

Skeletal muscle dysfunction is a common complication of critical illnesses such as acute respiratory distress syndrome (ARDS), leading to long-term muscle weakness in survivors (Herridge and Azoulay, 2023; Palakshappa et al., 2021). Bedridden patients with chronic lung disease or prolonged inactivity often experience skeletal muscle atrophy. Muscle atrophy in ARDS patients is caused by inflammation and disuse, with histological features including atrophy of type II muscle fibers, partial loss of myosin, and infiltration by immune cells (Files et al., 2012; Puthucheary et al., 2013).This muscle wasting contributes to impaired physical function and reduced capacity for muscle recruitment, which may further compromise respiratory muscles and immunity (Puthucheary and Prescott, 2017). This may subsequently exacerbate systemic inflammation.

##### 6.1.2.2 Pulmonary dysfunction leading to cardiovascular decline

**FIGURE 1:**
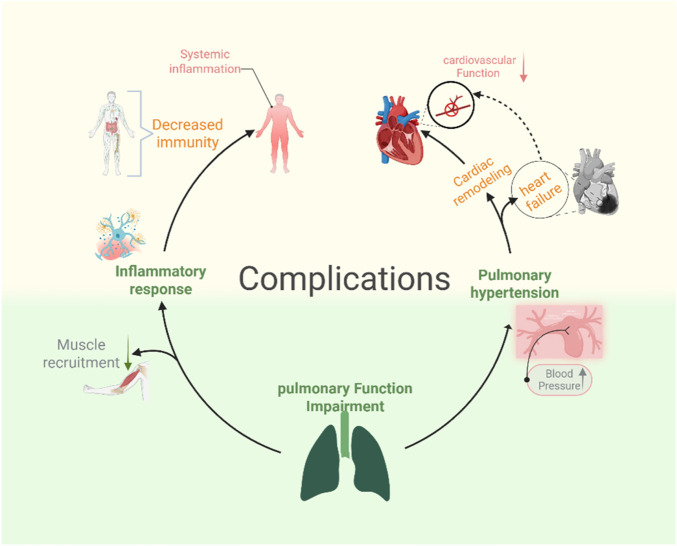
Some complications caused by pulmonary function impairment.

The hemodynamic coupling between the heart and lungs is the physiological basis for the cardiovascular decline caused by pulmonary dysfunction (Alvarado and Pinsky, 2023). Pulmonary capacity changes and hypoxemia resulting from lung damage can lead to cardiovascular failure. The interactions between the lungs and heart are continuous and dynamic, with fluctuations in lung volume, chest pressure, and oxygen levels affecting the cardiovascular system (Hamahata and Pinsky, 2023). These cardiovascular issues include pulmonary hypertension-induced cardiac remodeling and heart failure (Cassady and Ramani, 2020; Balistrieri et al., 2023; Fisher et al., 2022). The mechanisms described above are presented in Figure 1.

### 6.2 Exercise as core long-term management: constructing a multidimensional intervention system

#### 6.2.1 Community and home-based rehabilitation: technology-enabled remote rehabilitation systems

Using mobile medical rehabilitation platforms is safe and increasingly popular (Diamond et al., 2021). The advent of mobile health technologies (mHealth) has made long-term exercise interventions more accessible. Wearable smart devices actively track exercise parameters and metabolic states (Lu et al., 2020), and some mobile apps even provide suitable exercise prescriptions for frail elderly individuals (Soto-Bagaria et al., 2023). AI-powered wearable devices (Malik et al., 2024) enable multi-modal physiological monitoring → AI deep learning analysis → intelligent early warning systems, making it possible to monitor and provide real-time alerts for remote rehabilitation exercise interventions.

#### 6.2.2 Synergistic intervention of mind-body interaction

Cognitive behavioral therapy (CBT) helps alleviate emotional and psychological distress, improves distorted cognition, and establishes functional and balanced cognitive patterns (Naeem et al., 2021; Stallard, 2022). Combining exercise with CBT can reduce anxiety and depression caused by long-term sequelae while promoting lung function recovery and improving physical fitness (Zeraatkar et al., 2024; Scott et al., 2023). This integrated approach helps regulate both psychological and physiological aspects, leading to comprehensive rehabilitation.

#### 6.2.3 Safety and clinical stage-based stratification of exercise interventions

The core elements of exercise intervention safety and clinical staging primarily encompass the following three aspects: 1) Stratification of exercise modalities according to clinical phases (acute/unstable, recovery/subacute, and chronic/long-term sequelae), with the implementation of intensity-titrated protocols tailored to each stage (Foster et al., 2017); 2) Comprehensive pre-participation screening and identification of absolute and relative contraindications (Thompson et al., 2013), coupled with the recommendation that exercise be conducted under appropriate professional supervision to ensure patient safety; 3) Integration of pragmatic monitoring strategies and predefined cessation criteria, notably the routine application of continuous peripheral oxygen saturation (SpO_2_) monitoring in patients susceptible to hypoxemia, with cautionary thresholds commonly set around 88% (Garcia-Talavera et al., 2023).

### 6.3 Stratified management for special populations

The characteristics of exercise interventions for special populations vary. Firstly, in elderly patients, when considering exercise training as a long-term management strategy, a comprehensive assessment of their physical condition should be conducted. Based on individual interests and physical status, personalized exercise programs should be designed. Psychological counseling is essential to reduce the fear of falls in elderly patients and to increase participation in group exercises, which can enhance social interaction (Zhong et al., 2024).In the pediatric population, the use of virtual reality (VR) interaction can make the entire exercise process more engaging by gamifying it. Virtual reality (VR) has been confirmed as a safe and effective new rehabilitation tool (Patsaki et al., 2023), and VR-based therapies have the potential to improve motor and functional skills across a wide range of age groups by inducing cortical reorganization and activating various neuronal connections (Ali et al., 2023). VR can effectively improve gait and balance (Ghai and Ghai, 2019).For individuals with disabilities, simple exercise interventions such as inspiratory muscle training (Okrzymowska et al., 2024) and tandem cycling (Goodlin et al., 2022) can be employed. The exercise interventions tailored to different special populations are presented in Table 1.

**TABLE 1:** Differences in intervention strategies for different special populations.

Special populations	Pulmonary function characteristics	Exercise tolerance	Primary exercise limitations	Specialized intervention strategies	Recommended types	References
Elderly (≥60 years)	Decreased lung compliance, reduced gas exchange capacity, and muscle weakness	Low to moderate	Dyspnea, sarcopenia, and significant cardiovascular burden	Use of walking aids and fall prevention measures	Low-intensity: walking, Tai Chi, yoga	Schneider et al. (2021), Schuliga et al. (2021), Budde and Skloot (2022)
Children (<18 years)	Narrow airways, prone to wheezing, immature respiratory control ability	Moderate	Poor motor coordination and susceptibility to fatigue	Incorporating VR motion-sensing games	Moderate-intensity: jogging, ball sports	Pearkao et al. (2025), Wu et al. (2023)
Persons With Different Types Of Disabilities	Depending on the type of disability	Depending on the type of disability	Possible mobility limitations	Personalized rehabilitation equipment assistance	Low to moderate intensity, personalized adjustments	M et al. (2022), Jaarsma et al. (2014), Flueck (2021)

## 7 The mechanism of action of exercise intervention

### 7.1 Physiological basis

#### 7.1.1 Three mechanisms of ventilation-perfusion (V/Q) matching

**FIGURE 2:**
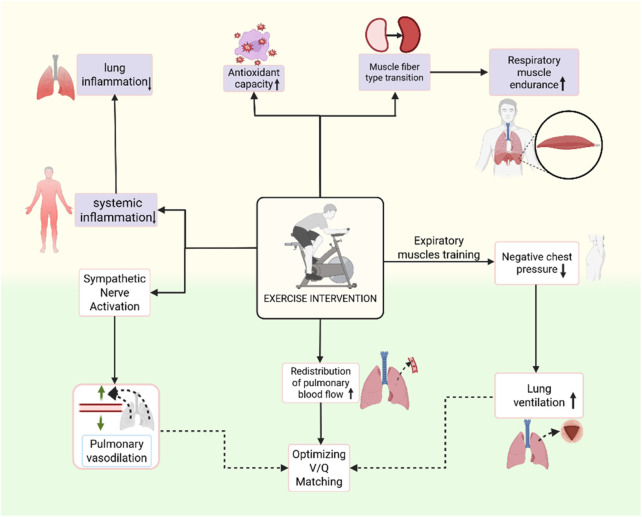
Mechanism mapping of exercise intervention in improving pulmonary function.

Pulmonary function impairment often leads to ventilation-perfusion mismatch (V/Q mismatch) (Squizzato et al., 2023), which affects gas exchange efficiency and results in hypoxemia. Exercise interventions optimize V/Q matching through three mechanisms: mechanical regulation, blood flow redistribution, and neurovascular regulation, thereby promoting lung function recovery. 1) Mechanical Regulation: Deep breathing training and inspiratory muscle training can increase chest cavity negative pressure, promote alveolar recruitment, and improve ventilation function (Miserocchi and Beretta, 2023). Endurance training can expand the chest cavity, increase diaphragm mobility, and enhance ventilation in the lower lungs. 2) Blood Flow Redistribution: Exercise enhances venous return and indirectly improves pulmonary capillary perfusion (Hebson et al., 2025; Kondo et al., 2018). Exercise interventions can also redistribute pulmonary circulation blood flow, improving overall perfusion efficiency. 3) Neurovascular Regulation: Exercise interventions activate the sympathetic nervous system, adjusting the sympathetic-parasympathetic balance to regulate bronchial tone (β_2_ receptor activation), promoting bronchial smooth muscle relaxation, and improving pulmonary ventilation (Zou et al., 2018; Jaenisch et al., 2017). Figure 2 reveals part of the mechanism.

#### 7.1.2 Reducing systemic inflammation

Patients with pulmonary function impairment often experience systemic inflammation, which not only further affects lung function but can also lead to other complications (Febbo and Dako, 2024). Exercise interventions regulate the immune system to alleviate inflammatory symptoms through the following mechanisms: 1) Exercise intervention lowers levels of pro-inflammatory factors such as IL-6, TNF-α, and IFN-γ (Sungurlu and Balk, 2024), while promoting the release of anti-inflammatory cytokine IL-10 to help reduce the inflammatory response (Almahayni and Hammond, 2024; Alves et al., 2022). 2) Short-term aerobic exercise increases the number of monocytes, neutrophils, and lymphocytes (Alawna et al., 2020), while long-term exercise intervention suppresses the excessive activation of pro-inflammatory M1 macrophages and promotes the proportion of anti-inflammatory M2 macrophages, thus improving chronic low-grade inflammation (Sleijser-Koehorst et al., 2023; Ghol et al., 2022). Short-term aerobic exercise temporarily increases circulating immune cells, while long-term regular aerobic exercise optimizes immune function and reduces chronic inflammation (Chastin et al., 2021; Salimans et al., 2022).

#### 7.1.3 Enhancing antioxidant capacity

Regular aerobic exercise activates the body’s antioxidant system, upregulating endogenous antioxidant factors and enhancing antioxidant enzyme activity (Ye et al., 2021). Exercise interventions also induce physiological adaptations that increase the activity of antioxidant enzymes, enabling muscles to resist oxidative stress (Duranti, 2023). A meta-analysis showed that regular practice of Tai Chi can increase the levels of superoxide dismutase and catalase (Rosado-Perez et al., 2021). Another study indicated that resistance training is associated with elevated levels of glutathione peroxidase (Cordeiro et al., 2025). Superoxide dismutase (SOD) (Zhao et al., 2021) and glutathione peroxidase (GSH-Px) (Flohe et al., 2022) are key antioxidant enzymes in the body, capable of scavenging free radicals and decomposing peroxides, thus reducing cell damage caused by oxidative stress and protecting lung function.

### 7.2 Structural adaptive changes

#### 7.2.1 Respiratory muscle remodeling

Exercise training can induce the transformation of some fast-twitch muscle fibers (Type II) into slow-twitch fibers (Type I) (Plotkin et al., 2021), enhancing the endurance characteristics of muscle fibers and improving the fatigue resistance of the diaphragm and intercostal muscles. Furthermore, exercise can increase muscle fiber cross-sectional area (hypertrophy), promoting diaphragm thickening, thereby enhancing the strength and endurance of respiratory muscles. Exercise also activates satellite cells, promoting the repair and growth of diaphragm and intercostal muscle fibers. Slow-twitch muscle fibers (Type I) in the lungs have higher fatigue resistance, and their increased proportion can improve respiratory muscle endurance (Del Corral et al., 2023), thereby improving lung function.

#### 7.2.2 Increased mitochondrial biogenesis in peripheral muscles

Exercise interventions promote mitochondrial biogenesis in peripheral muscles associated with lung function, with the following primary mechanisms: 1) Exercise increases the AMP/ATP ratio within cells, activating AMP-activated protein kinase (AMPK) and PGC-1α pathways, which promote mitochondrial biogenesis (Steinberg and Carling, 2019; Spaulding and Yan, 2022; Cai et al., 2023). 2) Moderate increases in reactive oxygen species (ROS) levels activate oxidative stress signaling and calcium (Ca^2+^)-dependent pathways (such as CaMK and p38 MAPK), facilitating mitochondrial adaptive remodeling (Powers et al., 2020; Qaisar et al., 2016; Arazi et al., 2021). 3) Exercise improves blood flow perfusion and oxygen supply to peripheral muscles (e.g., diaphragm, intercostal muscles, and skeletal muscles), enhancing aerobic metabolic capacity (Katayifci et al., 2022). 4) Exercise promotes mitochondrial autophagy (mitophagy), removing dysfunctional mitochondria and optimizing mitochondrial quality (Memme et al., 2021; Hood et al., 2019; Wu et al., 2019).These mechanisms together enhance the function of peripheral muscles related to lung function, improve overall metabolic capacity and endurance, boost exercise performance, and improve rehabilitation outcomes in patients with chronic lung diseases.

### 7.3 Applicable populations and disease stages for exercise intervention mechanisms

The exercise-induced mechanisms outlined above are chiefly derived from studies conducted on patients with impaired pulmonary function, including those in the acute phase (e.g., early rehabilitation following acute respiratory distress syndrome), recovery phase (post-discharge pulmonary rehabilitation), and chronic phase with persistent functional limitations (e.g., chronic obstructive pulmonary disease, pulmonary fibrosis); While physiological adaptations such as improved respiratory muscle strength, enhanced aerobic capacity, and optimized ventilation–perfusion matching are theoretically applicable to patients across different stages of pulmonary dysfunction, pathophysiological differences—such as airway obstruction, reduced lung compliance, and chronic hypoxemia—necessitate cautious extrapolation of findings from healthy populations. Therefore, when applying exercise prescriptions to clinical practice, intervention type, intensity, and monitoring requirements should be tailored according to the disease stage, with low-intensity, closely monitored training being more suitable for acute-phase patients, and progressive endurance–strength combined programs being appropriate for recovery and chronic-phase populations.

The stage-specific exercise intervention strategies presented in Table 2 can serve as a reference for exercise prescription design, highlighting the appropriate modalities tailored to each phase of pulmonary dysfunction.

**TABLE 2:** Pathological features and intervention options for each stage of pulmonary dysfunction.

Stage of pulmonary dysfunction	Pathological features	Exercise interventions	Evidence/Mechanisms	References
Acute Phase (e.g., during infection, post-exacerbation)	Acute inflammation, alveolar damage, hypoxemia	Passive/assisted mobilization, breathing exercises; very-low-intensity activity if stable (e.g., passive range of motion, progressive Inspiratory Muscle Training short supervised walk)	Improves oxygenation, prevents deconditioning	Rajan et al. (2023), G et al. (2018), Rochester et al. (2015), Ries et al. (2007), McCarthy et al. (2015)
Recovery Phase	Partial tissue repair, reduced lung compliance	Gradual aerobic (walking, cycling), light resistance	Enhances ventilatory efficiency, maintains muscle mass	
Stable/Chronic Phase	Fibrosis, airway remodeling, persistent airflow limitation	Aerobic + resistance training, Tai Chi, pulmonary rehab programs	Improves exercise tolerance, reduces dyspnea	
Late/Severe Stage	Extensive fibrosis/emphysema, hypoxemia at rest	Modified low-intensity training with oxygen therapy	Maintains functional independence, slows decline	

For patients in the acute phase, early mobilization should be planned based on assessment of cooperation, cardiopulmonary reserve, musculoskeletal function, and functional status (Spruit et al., 2013). It should adhere to progressive mobilization protocols (Stiller, 2013), and stop if SpO_2_ < 88% (Morris et al., 2008) or symptoms worsen.

## 8 Conclusion

In the comprehensive management of pulmonary function impairment throughout the entire disease course, from the acute phase to the long-term rehabilitation phase, exercise intervention offers clinical value that is difficult to fully replace with pharmacological treatments. As a safe and effective rehabilitative approach, it deserves widespread promotion and application. During the acute phase, moderate exercise intervention can improve pulmonary ventilation function, enhance oxygenation capacity, and promote systemic circulation (Stickland et al., 2013). Thereby reducing the risk of venous thromboembolism and potentially aiding in lung tissue repair. In the rehabilitation phase, scientific exercise training not only improves cardiopulmonary endurance and muscle strength (Posnakidis et al., 2022), but also enhances neuromuscular coordination (Sale, 1988), optimizes autonomic nervous function, and alleviates psychological stress responses such as anxiety and depression (Pearce et al., 2022), ultimately improving the overall quality of life for patients.

A comprehensive assessment of the patient’s health status, exercise preferences, and rehabilitation goals should guide the formulation of a scientific and personalized exercise strategy. Based on the “acute-transition-recovery-long-term” four-phase continuous exercise rehabilitation model, precise intervention can maximize the therapeutic benefits of exercise, promote lung function repair, improve long-term outcomes, and provide patients with a systematic rehabilitation pathway.

This study also highlights the current lack of scientifically validated stratification standards correlating exercise intensity with disease severity in pulmonary rehabilitation, as well as the significant limitation posed by poor long-term patient adherence. Future efforts should integrate precision medicine approaches, utilizing dynamic monitoring of multidimensional biomarkers to facilitate personalized exercise prescription formulation and adjustment. Concurrently, the application of advanced technologies such as artificial intelligence technique offers novel opportunities for real-time optimization of exercise protocols and patient management (Sonnerfors et al., 2025). These innovations hold promise to advance the scientific rigor, refinement, and individualization of pulmonary rehabilitation interventions, thereby improving clinical outcomes and patient quality of life.
